# Cyanuric Chloride with the s-Triazine Ring Fabricated by Interfacial Polymerization for Acid-Resistant Nanofiltration

**DOI:** 10.3390/membranes15080231

**Published:** 2025-08-01

**Authors:** Zhuangzhuang Tian, Yun Yin, Jiandong Wang, Xiuling Ao, Daijun Liu, Yang Jin, Jun Li, Jianjun Chen

**Affiliations:** 1 School of Chemical Engineering, Sichuan University, Chengdu 610065, China; 2 Engineering Research Center of Comprehensive Utilization and Clean Processing of Phosphorus Resources, Chengdu 610065, China

**Keywords:** phosphoric acid, interfacial polymerization, nanofiltration, acid resistance, selective separation

## Abstract

Nanofiltration (NF) is considered a competitive purification method for acidic stream treatments. However, conventional thin-film composite NF membranes degrade under acid exposures, limiting their applications in industrial acid treatment. For example, wet-process phosphoric acid contains impurities of multivalent metal ions, but NF membrane technologies for impurity removal under harsh conditions are still immature. In this work, we develop a novel strategy of acid-resistant nanofiltration membranes based on interfacial polymerization (IP) of polyethyleneimine (PEI) and cyanuric chloride (CC) with the s-triazine ring. The IP process was optimized by orthogonal experiments to obtain positively charged PEI-CC membranes with a molecular weight cut-off (MWCO) of 337 Da. We further applied it to the approximate industrial phosphoric acid purification condition. In the tests using a mixed solution containing 20 wt% P_2_O_5_, 2 g/L Fe^3+^, 2 g/L Al^3+^, and 2 g/L Mg^2+^ at 0.7 MPa and 25 °C, the NF membrane achieved 56% rejection of Fe, Al, and Mg and over 97% permeation of phosphorus. In addition, the PEI-CC membrane exhibited excellent acid resistance in the 48 h dynamic acid permeation experiment. The simple fabrication procedure of PEI-CC membrane has excellent acid resistance and great potential for industrial applications.

## 1. Introduction

The nanofiltration (NF) process is a well-established pressure-driven separation technique that exhibits high energy efficiency, excellent separation efficacy, and environmental friendliness, which is extensively used in wastewater treatment and product purification operations in chemical engineering, biotechnology, and pharmaceutical industries [1,2,3,4]. However, NF membranes used in industry require selective separation and stable operation performances under harsh environments (pH > 11 or pH < 2).

The strategy of using NF to remove a variety of metal ions (Fe^3+^, Al^3+^, Mg^2+^) in industrial phosphoric acid (H_3_PO_4_) production is both economical and environmentally friendly. However, this requires NF membranes to be able to reject metal ions in a broad range without affecting the permeation rate of H_3_PO_4_ molecules and have excellent acid resistance (pH < 1). Consequently, it is important to select suitable monomers for forming strong covalent bonds to construct acid-resistant and high-separation-performance NF composite films.

The most common commercial NF membrane is the polyamide (PA) membrane, which is stable only at pH > 2 due to the amide bond being easily hydrolyzed under extreme acid conditions [5,6]. Cyanuric chloride (CC) was employed to fabricate acid-resistant NF membranes via IP due to its relatively small molecular size, good solubility in the organic phase, and attractive thermal and chemical stability. CC features an s-triazine ring with a conjugated structure characterized by alternating carbon–nitrogen single and double bonds, conferring intrinsic acid resistance and stability. Consequently, the triazine–polyamine film formed through the reaction of CC with amine monomers provides exceptional acid resistance for nanofiltration membranes. Crucially, unlike polyamide membranes, the triazine–polyamine layer configuration lacks carbonyl groups, thereby avoiding susceptibility to protonation and resistance to nucleophilic attacks, which significantly enhances its acid resistance [7,8,9]. Selecting suitable monomers can adjust the surface charge of NF membranes to achieve selective ion permeation. Polyethyleneimine (PEI) is an excellent monomer for preparing positively charged NF membranes [10]. The functional layer containing many unreacted primary and secondary amines is formed by interfacial polymerization [11,12]. The amine groups would be protonated in acidic and neutral solutions, producing a strongly positively charged surface for rejecting multiple impurity ions. Khaless et al. employed PEI-functionalized nanofiltration membranes to achieve highly selective separation of metal ions via electrostatic repulsion between positively charged surfaces and metal cations under strongly acidic conditions [13].

Lasisi et al. demonstrated that the NF membrane synthesized from branched PEI and CC maintained a MgCl_2_ rejection above 80% after immersion in 20 wt% H_2_SO_4_ for 744 h [14]. Yu et al. further verified that the PEI-CC membrane exhibited only a 2.1–5.2% loss in MgCl_2_ rejection after 72 h exposure to 3 wt% HCl at 50 °C, significantly outperforming commercial polyamide membranes [7]. Bai et al. confirmed that the PEI-CC membrane retained a MgCl_2_ rejection of 96.34% after 60 days of immersion in 0.1 mol·L^−1^ HNO_3_ [15]. While the PEI-CC membrane maintains efficient separation performance in sulfuric acid, hydrochloric acid, and nitric acid environments, application studies in phosphate systems have not yet been reported.

To address the demand for removing metal impurities from high-concentration phosphoric acid solutions, this study innovatively introduces an acid-resistant PEI-CC membrane. Through orthogonal experimental design, precise modulation of the membrane microstructure is achieved. This research focuses on elucidating the selective separation mechanisms in environments with coexisting multi-metal ions (Fe^3+^, Al^3+^ and Mg^2+^), providing an efficient separation strategy for phosphoric acid purification. The highly acid-resistant NF membranes are fabricated by interfacial polymerization of PEI and CC with the s-triazine ring in this work. Additionally, acid-resistant porous polyethersulfone (PES) ultrafiltration membranes are used as support materials. The optimal conditions for PEI-CC membrane preparation were determined by L_16_ (4^5^) orthogonal experiments. The chemical composition and surface morphology of the membranes were systematically characterized by ATR-FTIR, XPS, AFM, SEM, and zeta potential. The average pore size and pore size distribution were investigated using PEG permeation.

## 2. Materials and Methods

### 2.1. General Materials

Polyethyleneimine (PEI, ~1800 Da, >99% purity) and cyanuric chloride (CC, 99% purity) were acquired from Aladdin. *N*-hexane (≥97%), sodium dodecyl sulfate (SDS), sodium hydroxide (NaOH), magnesium sulfate (MgSO_4_), aluminum sulfate (Al_2_(SO_4_)_3_), ferric sulfate (Fe_2_(SO_4_)_3_), and synthesis quality polyethylene glycol (PEG) with average molecular weights of 200, 400, 600, 800, 1000, 2000, 4000, 6000, 8000, and 10,000 g mol^−1^ were purchased from Kelong Chemical Co., Ltd. (Sichuan, China). Polyethersulfone (PES) ultrafiltration membrane with a molecular weight cut-off of around 8000 Da was provided by Risingsun Membrane Technology Co., Ltd. (Beijing, China). All the chemical reagents used in the experiments were of analytical grade and used directly without further purification. All aqueous solutions were prepared with deionized water.

### 2.2. Membrane Fabrication

**Figure 1:**
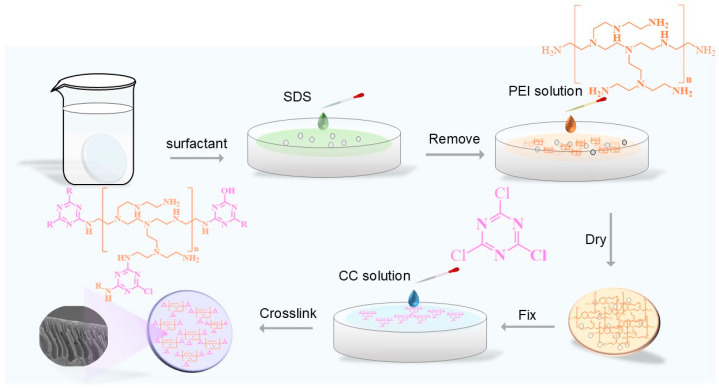
Schematic drawing of interfacial polymerization PEI-CC membrane. Five factors and four levels of orthogonal experiments were used to optimize the nanofiltration membrane fabrication conditions.

NF membranes were fabricated via interfacial polymerization of polyethyleneimine (aqueous phase) and cyanuric chloride (organic phase) on PES membranes, as illustrated in Figure 1. Firstly, the PES membrane was dipped into deionized water for 24 h to remove the residual impurities in the membrane. Then the membrane was sandwiched in a homemade interface polymerization device [6]. Next, the mixed solution of 15 mL SDS and NaOH (1 wt%) was poured on top of the PES membrane and allowed to stand for at least 10 min before draining the excess solution. This step serves to increase the wettability of the PES membrane. Subsequently, the membrane was put into the 15 mL PEI solution for 4.5 h, and the residual solution was drained. Afterward, the 15 mL organic solution containing CC was poured on top of the PES membrane for 5 min, and the solution was removed from the surface. In the final step, the membrane was stored in a drying oven to cure. Lastly, the fabricated NF membrane was immersed in deionized water for at least 24 h before being used. Table 1 shows the orthogonal experimental parameters.

### 2.3. Characterization

Scanning electron microscopy (SEM). A field-emission scanning electron microscope (Nova NanoSEM450, Hillsborough, OR, USA) was utilized to observe the morphology of the membrane surface. The accelerating voltage used for the observation was 5.0 kV.

Atomic force microscopy (AFM). Atomic force microscopy (Icon, Bruker, Germany) was applied to measure the surface roughness. The roughness was determined with a scanning area of 4.0 × 4.0 μm^2^.

Attenuated total reflectance–Fourier transform infrared spectroscopy (ATR-FTIR). The chemical properties of the membrane were analyzed using attenuated total reflectance infrared spectroscopy (Invenio R, Bruker, Germany).

Liquid contact angle (CA). The dynamic water contact angle (WCA) of the membranes was assessed using a drop shape analysis system (DSA25S, KRüSS GmbH, Hamburg, Germany).

X-ray photoelectron spectroscopy (XPS). X-ray photoelectron spectroscopy (XPS) (XSAM800, Kratos Co., Manchester, UK) was conducted to study the percentage content of elements on the membrane surface. Broad-survey XPS spectra were scanned by sweeping in the range of electron binding energy from 0 to 1400 eV using a 0.9 eV resolution. The narrow spectrum varies according to different elements. All of the membrane samples were vacuum-dried before XPS measurement.

Zeta potential. The membrane’s zeta potential was investigated using an electrokinetic analyzer (SurPASS, Anton Paar GmbH, Graz, Austria). For each experiment, two membranes of 20 mm × 10 mm were fixed on the adjustable gap cell of the electrodynamic analyzer by double-sided tape. The gap between the two membranes was constantly adjusted to a constant value of ~100 μm. Streaming current was measured in 1 mM KCl solution at 25 °C by Ag/AgCl electrodes mounted at the electrolyte inlet and outlet of the measuring cell and attached very close to the rectangular slit formed by the membranes. The pH of the KCl solution was adjusted by adding 0.05 M HCl and 0.05 M NaOH. For each pH point, four measurements were conducted.

The zeta potential was calculated according to the following equation:

(1) $$\zeta =\frac{dI}{dP}\frac{\eta }{\varepsilon \varepsilon_{0}}\frac{L_{s}}{A_{s}}$$

where ζ is the zeta potential [V], dI/dP is the gradient of streaming current versus pressure [A Pa^−1^], η  is the electrolyte dynamic viscosity [Pa s], ε is the dielectric constant of the electrolyte, $\varepsilon_{0}$ is the vacuum permittivity [F m^−1^], $L_{s}$ is the length of the streaming channel [m], and $A_{s}$ is the cross-section area of the streaming channel [m^2^] [16].

Molecular weight cut-off and pore size. The rejection of PEG aqueous solution with various molecular weights of 200, 400, 600, 800, 1000, 2000, 4000, 6000, 8000, and 10,000 Da was determined by the cross-flow filtration flat-sheet membrane module under 0.6 MPa pressure, and the MWCO curve was plotted. The PEG concentration after iodine complexation was determined using a UV–Visible spectrophotometer (Mapada, P5, Shanghai, China) [17]. The MWCO of the NF membrane can be obtained from the MWCO curve, defined as the molecular weight rejected by the membrane when the rejection of the solute in the feed solution reaches 90%. The pore radius distribution of the membrane can be expressed as follows [18]:

(2) $$\frac{dR(r_{p})}{dr_{p}}\;=\;\frac{1}{r_{p}ln\sigma_{p}\sqrt{2\pi }}exp\left[-\frac{{\left(lnr_{p}\;-\;ln\mu_{p}\right)}^{2}}{2{\left(ln\sigma_{p}\right)}^{2}}\right]$$

where $r_{p}$ stands for the pore radius; $\mu_{p}$, the mean effective pore radius, is the geometric mean radius of the solute when the rejection equals 50%; and $\sigma_{p}$, the geometric standard deviation of the membrane, is the ratio of solute Stokes radii at 84.13% and 50% rejection [19].

For PEG, the Stokes radius of the solute used can be calculated according to the following equation [20]:

(3) $$r_{s}\;=\;16.73\;\times \;10^{-3}\;\times {\;M}^{0.557}$$

where M stands for the molecular weight of the solute.

**Figure 2:**
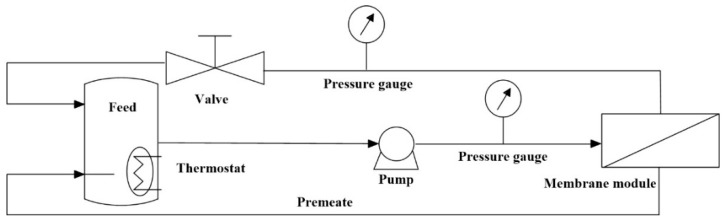
The cross-flow filtration set-up for determining the performance of the membranes.

Membrane performance test. The separation performance of the NF membrane was evaluated by employing a cross-flow filtration device with a circular filtration cell having an effective permeable membrane area of 6.88 cm^2^. A schematic diagram of the membrane filtration experiment is shown in Figure 2. The volumetric flow of the feed solution during performance testing was maintained at 7.0 L/h. The solution containing 2 g/L Mg^2+^, 2 g/L Fe^3+^, and 2 g/L Al^3+^ was used as the circulating solution of the cross-flow filtration device. The pH and P_2_O_5_ contents of the circulating solution were adjusted by adding 85% hot-process phosphoric acid. Before each permeation test, the membrane loaded in the filtration cell was pressurized at 0.8 MPa with the solution for 2 h to ensure a stable membrane. After that, the pressure of the filtration device was adjusted to 0.7 MPa to collect permeation. The permeation and feed concentrations of elements were measured by inductively coupled plasma optical emission spectroscopy (ICP-OES) (Optima 7000DV, Perkin Elmer, Hopkinton, MA, USA). Before ICP analysis, samples were diluted with deionized water and acidified with 10 mL of 33% HNO_3_. The rejection of the nanofiltration membrane was calculated according to the following equation:

(4) $$R_{i}\;=\;(1\;-\;\frac{C_{pi}}{C_{fi}})\times 100$$

where $C_{pi}$ and $C_{fi}$ represent the concentration given by ICP measurements in the permeation and feed, respectively. And i stands for elements such as Fe^3+^, Al^3+^, Mg^2+,^ and H_3_PO_4_.

To test the acid stability of the PEI-CC membranes, 20% phosphoric acid containing 2 g/L Fe^3+^, 2 g/L Al^3+^, and 2 g/L Mg^2+^ was used to continuously evaluate the membranes by the cross-flow device at 0.7 MPa for 48 h. Samples were taken every 6 h and analyzed for the concentration of each ion in the samples. The rejections of Fe^3+^, Al^3+^, and Mg^2+^, and phosphoric acid, as well as the flux, were used as quantitative measures of membrane stability.

## 3. Results and Discussion

### 3.1. Effect of Fabrication Conditions on Membrane Performance

**Figure 3:**
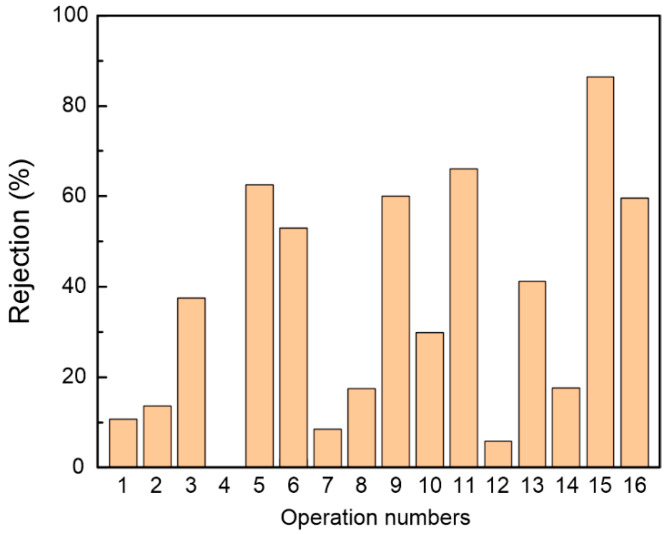
The results of orthogonal experiments calculated according to the total ion rejection of Fe^3+^, Al^3+^, and Mg^2+^.

All tests were conducted under the following conditions: The solution with 2 g/L Mg^2+^, 2 g/L Fe^3+^, and 2 g/L Al^3+^ was adjusted to a pH of 1 by adding phosphoric acid. The system pressure and temperature were 0.7 MPa and 25 °C, respectively. The optimal experimental conditions for fabricating PEI-CC membrane to reject Mg^2+^, Fe^3+^, and Al^3+^ in the solution were determined through the five-factor and four-level orthogonal experiments. As shown in Table 1, a total of 16 experiments were conducted. Each experiment was repeated once to ensure the accuracy of the experimental results. Figure 3 shows the total rejection of Mg^2+^, Fe^3+^, and Al^3+^ tested by each membrane in the orthogonal experiment. Obviously, N15 exhibited a significantly higher rejection rate than the others, reaching 86.46%. The experimental conditions of the N15 were a PEI of 40 g/L, a CC of 0.3 g/L, an SDS of 0.5 wt%, a curing temperature of 95 °C, and a curing time of 5 min. According to Table 1 and Figure 3, it can be found that when the ratio of PEI concentration to CC concentration is less than 100, the corresponding membrane rejection is relatively poor. This observation could be attributed to the reaction between PEI and CC occurring in the *n*-hexane phase [21,22]. When the two monomer solutions were in contact, PEI entered the *n*-hexane side through the liquid–liquid interface and reacted with CC. Therefore, a large excess of PEI over CC needed to be used, which drove PEI partitioning and diffusion into the organic phase. Any factors that changed the solubility and diffusivity of PEI in the organic phase would affect the reaction and thus the structure and morphology of the resulting PEI-CC membrane, which ultimately defined the separability and interfacial properties [23].

The results of the orthogonal experiment analysis for total ion rejection are shown in Table 2. The R values for PEI concentration, CC concentration, SDS concentration, curing temperature, and curing time were 0.3530, 0.2848, 0.2700, 0.3968, and 0.0261, respectively. Therefore, the degree of influence of these factors in descending order was curing temperature, PEI concentration, CC concentration, SDS concentration, and curing time. K_P4_ was the largest, indicating that a PEI concentration of 40 g/L was the most suitable for obtaining high total ion rejection. Similarly, K_C3_, K_S1_, K_T4_, and K_t4_ were the largest, indicating that 0.3 g/L CC, 0 wt% SDS, 95 °C, and 20 min were the optimal conditions for achieving high total ion rejection. However, it can be seen from the K value in Table 2 that the K value was still increasing when PEI concentration and curing temperature reached the upper limit in the experiment. Therefore, it was necessary to further design experiments on PEI concentration and curing temperature to explore the best NF membrane fabrication conditions.

Table 2 shows that the influence of CC concentration on NF membrane rejection exhibits a non-monotonic trend, initially decreasing and then increasing. This trend is mainly related to changes in the ratio of PEI concentration to CC concentration. In the process of NF membrane fabrication, CC was primarily used as a cross-linking agent. And its active chlorine reacted with the amine of PEI to generate a dense layer. Too low a CC concentration could lead to defects in the dense layer of the membrane. However, when the concentration of CC is too high, there would be a large amount of unreacted -Cl in the dense layer, resulting in more negative groups such as -OH being hydrolyzed from -Cl on the membrane surface, which would reduce the membrane surface’s positive charge density and lead to the lower rejection of metal ions [7].

SDS is an anionic surfactant with a hydrophilic head and hydrophobic tail. The SDS molecule and PEI were likely to form a micellar complex in the aqueous solution. It can be seen from Table 2 that the membrane rejection showed an apparent downward trend with the increasing SDS concentration. By increasing the concentration of SDS, the polar part of the surfactant molecule forms micelles with non-polar groups inside the surface and polar groups outside the micelles. With the presence of free micelles and the accumulation of polar groups in the polymer structure, the consequent increment in free volume in the thin layer led to the disruption of its structure [24].

### 3.2. Effect of PEI Concentration and Curing Temperature

**Figure 4:**
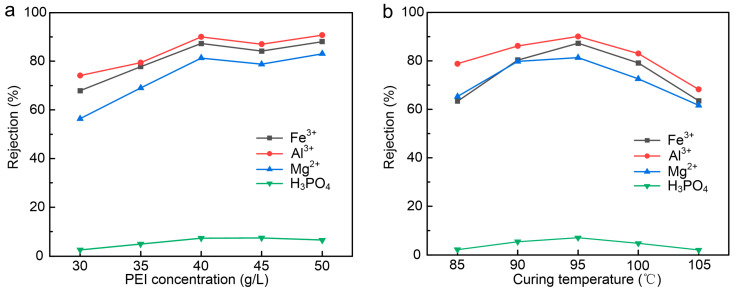
(**a**) Effect of the PEI concentration on the rejection. (**b**) Effect of the curing temperature on the rejection.

Continuing to optimize the PEI concentration, membranes were modified by the polymer in the range of 30–50 g/L. Other experimental conditions were maintained at the optimal values determined by the orthogonal experiments. Figure 4a shows the relationship between PEI concentration and the separation performance of the PEI-CC membrane. When the concentration of PEI increased from 30 g/L to 40 g/L, the rejection of Fe^3+^, Al^3+^, and Mg^2+^ increased by 19.4%, 15.91%, and 24.95%, respectively. However, when the PEI concentration increased from 40 g/L to 50 g/L, the rejection of the NF membrane changed minimally. The rejection of H_3_PO_4_ by the NF membrane follows the same trend as that of the metal ions.

At low concentrations of PEI solution, the interfacial polymerization process was expected to be slow, leading to a looser selective layer that could not effectively reject metal ions. At this time, the separation performance of the positively charged nanofiltration membrane was mainly affected by steric hindrance. With the increase in PEI concentration, the functional layer formed by interfacial polymerization became denser, so the rejection of ions increased. However, when the concentration of PEI reached 40 g/L, increasing the concentration had little effect on the rejection. This is because when the functional layer was well formed, the separation performance of the positively charged NF membrane was dominated by the Donnan exclusion theory.

To optimize the curing temperature, membranes were modified at curing temperatures in the range of 85–105 °C. Other experimental conditions were the optimal conditions determined by orthogonal experiments. Figure 4b shows the relationship between curing temperature and rejection performance of the PES-CC membrane. When the curing temperature increased from 85 °C to 95 °C, the rejection of Fe^3+^, Al^3+^, and Mg^2+^ increased by 23.9%, 11.23%, and 16.05%, respectively. Heat curing helped remove the residual organic solvent and promoted additional cross-linking of the functional layer. The molecule of CC had three chlorines, which could be substituted by amines and other nucleophilic substances step by step. The stepwise manner could be controlled at a well-defined temperature. Nevertheless, the substitution form also depended on the structure of the nucleophile, which was its basic strength and steric factors, the substituent already existent in the s-triazine ring, and the solvent used. In general, the substituent rule of chlorine in the CC is that monosubstitution occurs below or at 0 °C, disubstitution occurs at room temperature, and triple substitution occurs above 60 °C [25,26]. Therefore, the higher the curing temperature was, the more beneficial the reaction was for the reaction between PEI and CC. However, exposure to excessive temperature would destroy the structure of the functional layer. As shown in Figure 3, when the curing temperature increased from 95 °C to 105 °C, the rejection of Fe^3+^, Al^3+^, and Mg^2+^ decreased by 15.59%, 21.78%, and 19.74%, respectively. According to the test, we believe that the PEI/CC membrane prepared at 95 °C exhibited excellent performance. Therefore, the optimal conditions for NF membrane production were 40 g/L PEI, 0.3 g/L CC, 0 wt% SDS, a curing temperature 95 °C, and a curing time of 20 min.

### 3.3. Surface Morphology and Composition

**Figure 5:**
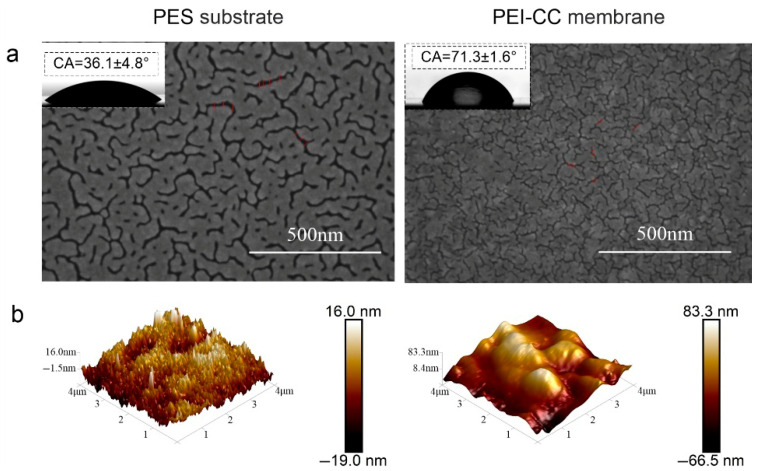
(**a**) Water contact angle and SEM of PES substrate and PEI-CC membrane of (**b**) AFM surface morphologies of PES substrate and PEI-CC membrane.

The surface morphology of the PEI-CC membrane was characterized by SEM and AFM, and the obtained images were compared with the PES substrate. As shown in Figure 5a, pores can be observed on the surface of the PES substrate, which are uniformly distributed throughout the entire membrane. SEM analysis of the PEI-CC membrane revealed that interfacial polymerization produced numerous uniformly tiny nodules. These nodules formed through the diffusion of PEI into the organic phase, indicating the growth of a polyamine network on the PES substrate, ultimately creating a dense selective layer on top of the porous PES substrate.

The formation of a dense and continuous layer of polyamine activity is attributed to the successful interfacial polymerization between the aqueous PEI and non-aqueous CC solutions. In addition, the surface roughness of the bare PES substrate was relatively smooth compared to the PEI-CC membrane. The increase in surface roughness after deposition of the PEI/CC active layer indicates the formation of an active layer on the PES substrate. After deposition of the PEI/CC active layer, the surface roughness increased from Rq = 4.86 nm (PES) to Rq = 19.4 nm (PEI-CC). In order to investigate the surface hydrophilicity of the PEI-CC membrane, the WCA was measured and compared with that of the bare PES substrate. The WCA increased from 36.1° (PES) to 76.3° (PEI-CC), indicating the deposition of a polyamine active layer on the PES substrate. This result is close to the result reported in the literature (77.5°) [27].

The chemical structure of the PEI-CC and PES membranes was investigated by ATR-FTIR.

**Figure 6:**
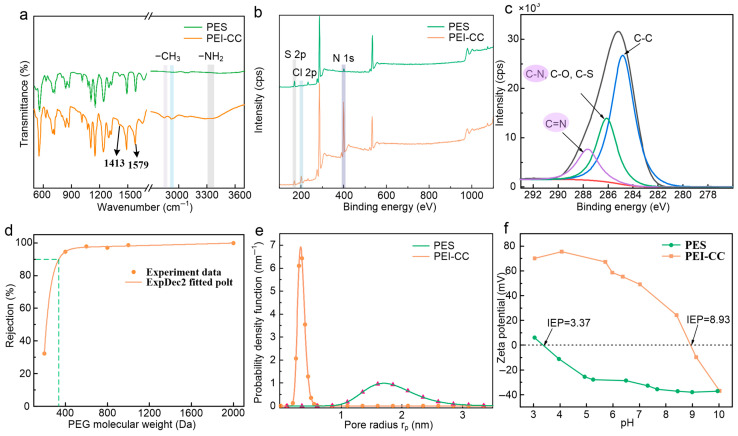
(**a**) ATR-FTIR spectra of the membranes, (**b**) XPS spectra of the PEI-CC membranes, (**c**) XPS high-resolution peak of carbon of the PEI-CC membranes, (**d**) PEG rejection curve for the PEI-CC membrane, (**e**) pore radius distribution of the PES substrate and PEI-CC membrane, and (**f**) surface zeta potentials of the PES substrate and PEI-CC membrane at different pH values.

As shown in Figure 6a, the PEI-CC membrane exhibits two new peaks at 2850 and 2926 cm^−1^, which are attributed to -CH [28]. A new peak appears in the range of 3200 to 3400 cm^−1^, indicating the presence of -NH_2_ and -NH [6]. Additionally, the peaks at 1413 and 1579 cm^−1^ are associated with the triazine ring present in the CC, indicating that PEI-CC was successfully synthesized [9,29].

In addition, the high-resolution XPS spectra were obtained to investigate the elemental composition of the fabricated membranes (Figure 6b,c). It was found that the peak intensity of N 1s of the PEI-CC membrane was significantly enhanced compared to those observed for the PES substrate, indicating that a large amount of N was introduced into the membrane surface, which existed in the amine group of the PEI and the s-triazine ring of the CC. Moreover, the small peak observed in 233.5 eV was attributed to S 2p. It can be seen that the peak intensity of S 2p of the PEI-CC membrane was weaker than that of the PES substrate because S was present only in the sulfone of the PES and not in the functional layer of the NF membrane. As shown in Figure 6c, the PEI-CC membrane had a peak at 287.61 eV, which was C=N. These indicated that PEI and CC had been successfully polymerized on the PES substrate surface.

Figure 6d shows the rejection curve of the PEI-CC membrane for PEG solutions with different molecular weights. When the PEG rejection reached 90%, the corresponding PEG molecular weight was approximately 337 Da, which fell in the range of conventional NF membranes. The pore radius of the membrane was 0.428 nm according to Equation (3) calculations. The results showed that the PEI-CC membrane effectively screened substances with a Stokes radius greater than 0.428 nm. Figure 6e illustrates the pore radius distribution of the PEI-CC membrane and PES substrate. The mean effective pore radius of the PEI-CC membrane was about 0.34 nm, much smaller than that of the PES substrate at 1.79 nm. The pore radius range of the PEI-CC membrane was between 0.18 and 0.61 nm, which was narrower than that of the PES substrate of 0.85–3.43 nm. It indicated that the interface polymerization of PEI/CC on the surface of the PES substrate formed a dense layer. The membrane pore radius was reduced, and the membrane pore radius distribution was more concentrated, which was more effective in rejecting ions with a smaller Stokes radius.

Figure 6f shows the zeta potential on the surface of the PES substrate and PEI-CC membrane measured by the flow potential in the pH range of 3–10. It was clearly shown in Figure 6f that the isoelectric point (IEP) of the PES substrate was about 3.37. When the pH was above 3.37, the PES substrate was negatively charged, which could be attributed to the deprotonation of high-density sulfonic groups. The isoelectric point of the PEI-CC membrane was about 8.93, indicating that the membrane was positively charged below a pH of 8.93. The PEI-CC membrane contained large amounts of secondary and tertiary amines that can be protonated. In addition, the PEI-CC membrane had s-triazine rings with nitrogen atoms, which can also be protonated and form positive groups. When the pH was above 8.93, the PEI-CC membrane was negatively charged owing to the unreacted chlorine group, which could hydrolyze at higher pH. The PEI-CC membrane had a positive charge in a more extensive pH range than the PES substrate. According to the Donnan exclusion, the membrane had an obvious advantage for rejecting multivalent metal ions in neutral and acidic solutions.

### 3.4. Separation Performance

In this study, the separation performance of the PEI-CC membrane was evaluated by a cross-flow filtration device. We adjusted the concentration of phosphoric acid in the solution and the temperature of the solution. We observed changes in the rejection rates to analyze the NF membrane’s performance.

**Figure 7:**
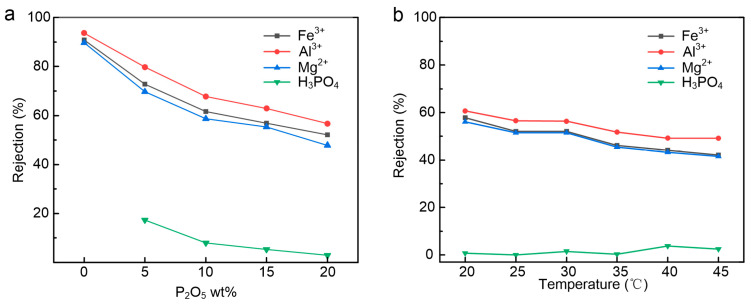
(**a**) The effect of P_2_O_5_ wt% on membrane performance; (**b**) the effect of temperature on membrane performance; the test conditions: solutions of 2 g/L Mg^2+^, 2 g/L Fe^3+^, 2 g/L Al^3+^, and varying of P_2_O_5_ solution.

The rejection of Fe^3+^, Al^3+^, Mg^2+^, and H_3_PO_4_ was mainly affected by steric hindrance, Donnan exclusion, and dielectric exclusion. The steric hindrance effect of the NF membrane on solutes depends on the membrane’s pore size and the solute’s radius. Donnan exclusion is determined by the surface charge density and properties of the NF membrane, as well as the charge characteristics of ions. The contribution of dielectric exclusion is proportional to the square of the ionic valence. It could be found from Figure 7a that the rejection of H_3_PO_4_ gradually decreased with the increase in phosphoric acid concentration. Table 3 shows that as phosphoric acid concentration increases, the solution pH decreases and the proportion of undissociated phosphoric acid molecules rises. When the phosphoric acid concentration increased from 5 wt% P_2_O_5_ to 10 wt% P_2_O_5_, the rejection of H_3_PO_4_ decreased from 17.32% to 7.87%. The reason was that the proportion of H_2_PO_4_^−^ in the 10 wt% P_2_O_5_ solution was smaller than that in the 5 wt% P_2_O_5_ solution. Compared with undissociated phosphoric acid molecules, the larger hydrated radius and negative charge of H_2_PO_4_^−^, along with the reduced positive charge on the membrane surface, resulted in the more likely rejection of H_2_PO_4_^−^. When the phosphoric acid concentration was greater than 10 wt% P_2_O_5_, most of the phosphoric acid was in the undissociated state, and its passage through the positively charged membrane was mainly controlled by steric hindrance. Therefore, the rejection of H_3_PO_4_ exhibited very little change. As shown in Figure 7b, the rejection of H_3_PO_4_ slightly increases with the increased temperature of the 20 wt% P_2_O_5_ solution. The increasing temperature was conducive to phosphoric acid dissociation because phosphoric acid dissociation was an endothermic process, resulting in a growing proportion of H_2_PO_4_^−^. Meanwhile, the pore size of the membrane increased with the temperature increase, which is conducive to the transmembrane transport of H_3_PO_4_. After these two factors partially canceled out, the rejection of P increased slightly.

Figure 7a shows the rejection curves of the PEI-CC membrane in solution with different phosphoric acid concentrations. Obviously, it could be found that the rejection of Fe^3+^, Al^3+^, and Mg^2+^ decreased with the addition of phosphoric acid and the increase in phosphoric acid concentration. When the phosphoric acid concentration in the solution increased from 0 to 20 wt% P_2_O_5_, the rejection of Fe^3+^, Al^3+^, and Mg^2+^ decreased from about 90% to 50%. The hydration radius of Fe^3+^, Al^3+^, and Mg^2+^ are 0.48 nm, 0.48 nm, and 0.428 nm, respectively. The pore radius of the nanofiltration membrane fabricated in the experiment was 0.428 nm, and the pore radius distribution range of the membrane was 0.18–0.61 nm. Therefore, ideally, the positive charge nanofiltration membrane had a higher rejection of Fe^3+^, Al^3+^, and Mg^2+^.

However, the experimental results showed that the NF membrane has a high rejection of Fe^3+^, Al^3+^, and Mg^2+^ only in the circumstance without phosphoric acid in the solution. The addition of phosphoric acid resulted in a significant decrease in the rejection of Fe^3+^, Al^3+^, and Mg^2+^. The following two factors might cause this. On the one hand, with the increased concentration of phosphoric acid, the ionic strength of the solution was high, and the double electric layer on the surface of the NF membrane was compressed and thinned. Moreover, the amount of H_2_PO_4_^−^ in the solution also increases, and these negatively charged ions can screen part of the positive charge on the surface [30,31]. Therefore, the positive charge of the nanofiltration membrane was reduced, resulting in a decrease in the repulsive force of cations. On the other hand, the active layer swelled due to the increase in ionic strength [32,33]. Therefore, the retention value decreased due to the increase in convective transport.

Figure 7b shows the PEI-CC membrane’s rejection curves in solution with different temperatures. It could be seen that the rejection of Fe^3+^, Al^3+^, and Mg^2+^ slowly decreased with the increase in temperature. The following two reasons may cause it: First, the pore size of the PEI-CC membrane increased with the increase in temperature. Secondly, the diffusion coefficient of the solute increased with increasing temperature, which made more ions pass through the membrane.

Figure 7 shows that the rejection increased in the order of Mg^2+^ < Fe^3+^ < Al^3+^, which basically conformed to the order of hydration radius and valence state of Fe^3+^, Al^3+^, and Mg^2+^ shown in Table 4. It is worth noting that Fe^3+^ and Al^3+^ had the same valence state and hydration radius, and the rejection of Al^3+^ was always greater than that of Fe^3+^. We compared the diffusion coefficients of Fe^3+^ and Al^3^ and obtained the following order for diffusion coefficients: Fe^3+^ > Al^3+^. Fe^3+^ had a higher diffusion coefficient than Al^3+^, so the rejection of Fe^3+^ was smaller than that of Al^3+^.

### 3.5. Acid Stability Evaluation of the Membrane

**Figure 8:**
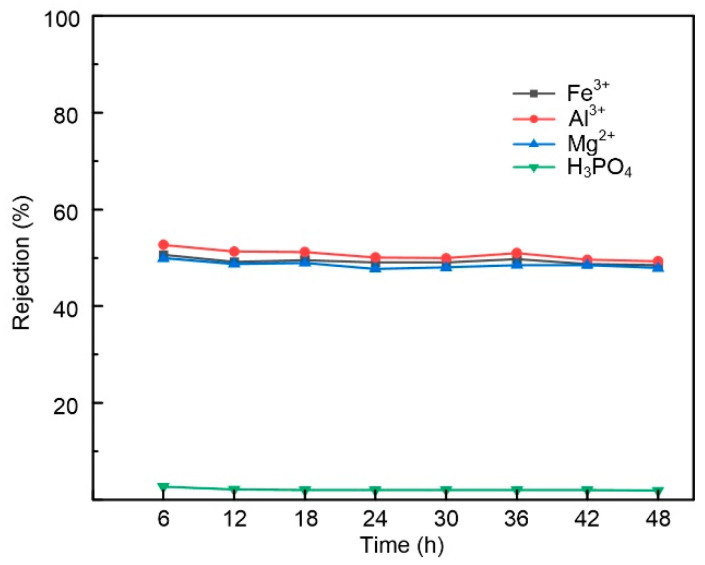
Variations in rejection and flux with time for the NF membrane in filtration aqueous solution containing 20 wt% P_2_O_5_, 2 g/L Fe^3+^, 2 g/L Al^3+^, and 2 g/L Mg^2+^ at 0.7 MPa and 25 °C.

To evaluate the stability of the PEI-CC membrane in phosphoric acid, the membranes were run continuously for 48 h in a solution containing 20 wt%P_2_O_5_, 2 g/L Fe^3+^, 2 g/L Al^3+^, and 2 g/L Mg^2+^. Figure 8 shows that the permeance and rejection of Fe^3+^, Al^3+^, Mg^2+^, and H_3_PO_4_ remained stable for 48 h, except that the permeance increased to some extent and the rejection decreased slightly in the first 6 h. It was probably caused by swelling of the functional layer in the membrane in highly concentrated phosphoric acid solutions in the first 6 h. However, the basic structure of the membrane remained intact and maintained good acid stability. The results indicated that the prepared PEI-CC membrane had good stability in phosphoric acid. The results are consistent with those reported in the literature [7,14,15]. The experimentally generated functional layer of the PEI-CC membrane with its s-triazine ring rigid structure is likely responsible for mitigating the initial nucleophilic attack on the membrane and enhancing the stability of acid. As shown in Table 5, the PEI-CC membrane exhibits a lower rejection of H_3_PO_4_ compared to reported data.

## 4. Conclusions

Acid-stabilized positively charged PEI-CC membranes for phosphoric acid purification were successfully prepared on the surface of the PES substrate by interfacial polymerization of PEI and CC. Orthogonal experiments determined the optimal conditions for the preparation of the PEI-CC membrane: 40 g/L PEI, 0.3 g/L CC, 0 wt% SDS, a curing temperature of 95 °C, and a curing time of 20 min. The isoelectric point of the NF membrane was around 8.93, and the MWCO was 337 Da. The membrane had good multivalent ion separation performance. The rejections of Fe^3+^, Al^3+^, and Mg^2+^ were in the order of Al^3+^ >Fe^3+^ >Mg^2+^. In addition, the membrane had a very low rejection of phosphoric acid, around 2.18% in 20% P_2_O_5_ solution. The membrane showed comparable rejection and permeance stability for a 20% phosphoric acid solution in the acid-stabilized test. Therefore, the membrane was expected to be applied in phosphoric acid recovery and purification.

## Figures and Tables

**Table 1:** The L16 (45) orthogonal table used for the study.

Operation Numbers	Factors				
	PEI (g/L)	CC (g/L)	SDS (wt%)	Curing Temperature (°C)	Curing Time (min)
N1	10	0.1	0	65	5
N2	10	0.2	0.5	75	10
N3	10	0.3	1	85	15
N4	10	0.4	1.5	95	20
N5	20	0.1	0.5	8	20
N6	20	0.2	0	95	15
N7	20	0.3	1.5	65	10
N8	20	0.4	1	75	5
N9	30	0.1	1	95	10
N10	30	0.2	1.5	85	5
N11	30	0.3	0	75	20
N12	30	0.4	0.5	65	15
N13	40	0.1	1.5	75	15
N14	40	0.2	1	65	20
N15	40	0.3	0.5	95	5
N16	40	0.4	0	85	10

**Table 2:** The results of the orthogonal experiment analysis for rejection.

	Factors				
	PEI (P)	CC (C)	SDS (S)	Temperature (T)	Time (t)
Σ(1)	0.6357	1.7436	1.8936	0.4260	1.4430
Σ(2)	1.4150	1.1400	1.6846	1.3837	1.4173
Σ(3)	1.6176	1.9860	1.3246	1.8934	1.3758
Σ(4)	2.0479	0.8467	0.8134	2.0131	1.4801
K1	0.1589	0.4359	0.4734	0.1065	0.3608
K2	0.3537	0.2850	0.4212	0.3459	0.3543
K3	0.4044	0.4965	0.3311	0.4733	0.3440
K4	0.5120	0.2117	0.2033	0.5033	0.3700
R	0.3530	0.2848	0.2700	0.3968	0.0261
Optimum condition	40 g/L	0.3 g/L	0 wt%	95 °C	20 min

**Table 3:** The existing forms of phosphoric acid and solution pH in different concentrations of phosphoric acid solutions.

	5 wt% P_2_O_5_	10 wt% P_2_O_5_	15 wt% P_2_O_5_	20 wt% P_2_O_5_
H_3_PO_4_ (%)	90.6	93.4	94.7	95.5
H_2_PO_4_^−^ (%)	9.4	6.6	5.3	4.5
pH	1.16	1.00	0.90	0.83

**Table 4:** Characteristics of the ions [34].

Ions	Bare Radius (nm)	Hydrated Radius (nm)	Hydration Number (±1)	Diffusion Coefficient (10^−9^ m^2^/s, 25 °C)
Fe^3+^	0.064	0.48	6	0.607
Al^3+^	0.053	0.48	6	0.559
Mg^2+^	0.072	0.428	6	0.705

**Table 5:** Comparison of membrane performance with reported data.

Membranes	Rejections of				Acid Stability/H_3_PO_4_	Rejection Decreased	Ref
	H_3_PO_4_	Fe^3+^	Al^3+^	Mg^2+^			
NF270	66%	/	/	93%	/	/	[35]
Desal-5 DK	33%	/	98%	/	0.4 mol/L (soak for 7 weeks)	≤5%	[36]
MPF34	~20% *	80–90%	/	/	/	/	[37]
MPF34	9.2	26.9	/	/	/	/	[38]
PDADMAC/PSS	/	/	/	65–75%	15 wt% (24 h)	≤5%	[30]
PDADMAC/PSS	5%	/	93%	/	10 wt% (60 min)	2%	[39]
PSS/PAH	10%	/	97%	/	10% (32 h)	<2%	[40]
This work	<3%	58%	60%	56%	20% P_2_O_5_ (48 h)	<5%	

* “~” indicates an approximate value.

## Data Availability

The original contributions presented in this study are included in the article. Further inquiries can be directed to the corresponding author.
